# Rare Movement Disorders—An Approach for Clinicians

**DOI:** 10.3390/ijms26136024

**Published:** 2025-06-23

**Authors:** Aaron Jesuthasan, Francesca Magrinelli, Amit Batla, Kailash P. Bhatia

**Affiliations:** 1 Department of Neurology, Addenbrooke’s Hospital, Cambridge CB2 0QQ, UK; a.jesuthasan@nhs.net; 2 Department of Clinical and Movement Neurosciences, UCL Queen Square Institute of Neurology, University College London, London WC1N 3BG, UK; f.magrinelli@ucl.ac.uk (F.M.); a.batla@ucl.ac.uk (A.B.)

**Keywords:** chorea, diagnosis, dystonia, genetics, parkinsonism, treatment

## Abstract

Rare genetic movement disorders usually manifest early in life with dystonia, parkinsonism, chorea, or a combination thereof. These are often associated with neurodevelopmental delay, intellectual disability, speech problems, retinal abnormalities, seizures, ataxia, spasticity, or systemic features. Due to their vast number and pheno–genotypic heterogeneity, the diagnosis of these disorders can be challenging. However, recognising their core motor phenomenology as well as clinical, laboratory, and neuroradiological clues can expedite appropriate diagnostic workup, molecular diagnosis, and adequate treatment. In this review, we outline diagnostic clues to rare movement disorders (RMDs), focusing on those that present mainly with dystonia, parkinsonism, or paroxysmal dyskinesia due to genetic causes. Additionally, we provide a decision tree approach linking clinical, genetic, and imaging testing. Finally, we highlight selected RMDs that should not be missed, as they possess established treatments that can hinder their progression, prevent irreversible or life-threatening sequelae and, in certain cases, lead to complete symptom remission.

## 1. Introduction

Rare movement disorders (RMDs) are genetic syndromes that often result from structural or functional abnormalities of the basal ganglia. They present with hypo- and/or hyperkinetic movements, either isolated or associated with other neurological and systemic manifestations. Although RMDs can also have an acquired aetiology (e.g., antibody-mediated), this review focuses on genetic RMDs according to the Orphanet definition of “rare diseases”, i.e., conditions affecting no more than 1 in 2000 individuals in Europe [1]. Using selected groups of RMDs as exemplars, this review aims to support the recognition of some key clinical features, alongside specific findings from laboratory tests and neuroimaging, that can assist neurologists in establishing an aetiological diagnosis and facilitating the timely initiation of further investigations or dedicated treatments.

Although most of the RMDs discussed in this review are individually very rare, they collectively represent a significant proportion of referrals to movement disorder specialists. Far from being exhaustive, this review focuses on selected monogenic RMDs grouped according to their underlying pathological mechanisms, including brain mineralisation disorders, lysosomal storage disorders, episodic disorders, vitamin deficiency-related disorders, and dopamine responsive disorders. Despite their diverse aetiologies, all of these disorders disrupt the neural pathways that regulate movement control.

## 2. General Clinical Approach to RMDs

The diagnosis of RMDs is often challenging as different disorders share similar phenotypic manifestations (genotypic heterogeneity), and many RMDs lack pathognomonic features or diagnostic biomarkers [2]. Furthermore, the clinical presentation may differ from the known core disease phenotype (phenotypic heterogeneity) [3]. A movement disorder specialist is often best placed to formally diagnose an RMD, given their clinical expertise and experience in the field [4]. Accordingly, referral to a specialist centre is appropriate once an RMD is suspected in order to expedite diagnosis, ensure a comprehensive diagnostic workup, establish the molecular diagnosis, and initiate specific treatments. Such settings also allow for the creation of a multidisciplinary team that can provide highly specialised input on diagnosis and treatment. However, general neurologists should remain vigilant for certain clinical clues and findings from investigations that can help establish a diagnosis. This is particularly relevant for patients in resource-limited settings, where access to specialist care may be significantly delayed [5]. In such cases, it is essential that a basic neurological history, clinical examination, and initial investigations are conducted to avoid missing time-critical treatments.

The age of symptom onset can be a helpful diagnostic clue, with half of all RMDs manifesting in childhood [6]. Nevertheless, genetic disorders typically associated with a childhood onset may still occasionally present later in life and vice versa. For example, *PLA2G6*-associated neurodegeneration most often presents with neuroaxonal dystrophies in early childhood but can also sometimes manifest with dystonia–parkinsonism in late adolescence or early adulthood [7,8]. In contrast, while Huntington’s disease usually presents in mid-adulthood with generalised chorea as the core motor feature [9], akinetic–rigid syndrome (Westphal variant) may also occur as a juvenile presentation of the disease [10].

A detailed clinical history is instrumental as an initial step to establish an appropriate differential diagnosis in RMDs. Developmental history and details of perinatal history are important. As most RMDs display a variety of neurological manifestations, some with intellectual impairment and spasticity, cerebral palsy is often a consideration [11]. In such situations, a detailed timeline of events, including the onset and progression of each symptom, can help to determine a progressive course that would favour an RMD or non-progressive sequelae of a perinatal insult in cerebral palsy [12]. Past medical and family history are vital to further indicate a possible diagnosis. For example, a personal or family history of epilepsy, hearing impairment, or myoclonus may suggest a mitochondrial disorder [13,14]. Constructing a good pedigree can assist in identifying a mode of inheritance and refining the differential diagnosis. Notwithstanding, an unremarkable family history should not prevent efforts to establish a molecular diagnosis if there is a high index of suspicion of an RMD, particularly in view of the frequent occurrence of incomplete penetrance in autosomal dominant disorders [15,16], compound heterozygosity in autosomal recessive disorders, and intrafamilial phenotypic heterogeneity [2].

By recognising movement disorders as either “isolated” when occurring alone or “combined” when associated with other movement disorders, non-motor neurological features or systemic manifestations (suggestive of an underlying syndrome) can point towards specific aetiologies [17]. Following the taking of clinical history, a comprehensive clinical examination remains imperative to facilitate an accurate diagnosis of an RMD. For instance, among early-onset dystonia syndromes, the detection of isolated generalised dystonia will first raise the suspicion of *TOR1A*- or *THAP1*-related dystonia [18,19], whereas the co-occurrence of myoclonus will suggest mutations in *SGCE*, *ANO3*, or *KCTD17* [20,21,22]. A thorough examination may also reveal pathognomonic or highly typical signs, such as Kayser–Fleischer rings in Wilson’s disease or the forceful sensory geste so-called “mantis sign” in pantothenate kinase-associated neurodegeneration (PKAN) (Table 1) [23,24].

Targeted investigations would be contingent on the information gathered and overall suspicions from the clinical history and examination, but may include biochemical tests (Table 1), searching for elevated plasma creatine kinase in neuroacanthocytosis or raised plasma/CSF lactic acid levels in mitochondrial disorders [25,26,27], low and absent plasma ceruloplasmin in Wilson’s disease and aceruloplasminemia, respectively, increased urine copper in Wilson’s disease, raised plasma oxysterols in Niemann–Pick disease type C (NP-C) [28], a reduced CSF-to-blood glucose ratio in GLUT1 deficiency syndrome, and abnormal CSF catecholamine metabolite levels in dopamine synthesis disorders [29].

Neuroimaging may also be pursued and is often crucial in the diagnostic workflow, as there may be particular findings which are highly suggestive of a specific RMD. For instance, low MRI-SWI signal from the basal ganglia and dentate nuclei corresponding to CT hyperdensity implies a brain calcification disorder [30], while if the CT is normal, it suggests iron deposition [31]. The latter can present with a typical pattern, such as the “eye of the tiger” sign that highly suggests but is not pathognomonic of PKAN [32]. The finding of MRI-T1 hyperintensity in the basal ganglia is another example, detected in disorders with manganese deposition in the brain [33].

Genetic testing may be considered based on the clinical history and examination alone or following suggestive investigation findings to reach a molecular diagnosis. A dopamine trial is also recommended for all patients with suspected RMD without a clear diagnosis, as dopa-responsiveness is a very helpful clue to elucidate the correct diagnosis [6].

**Figure 1:**
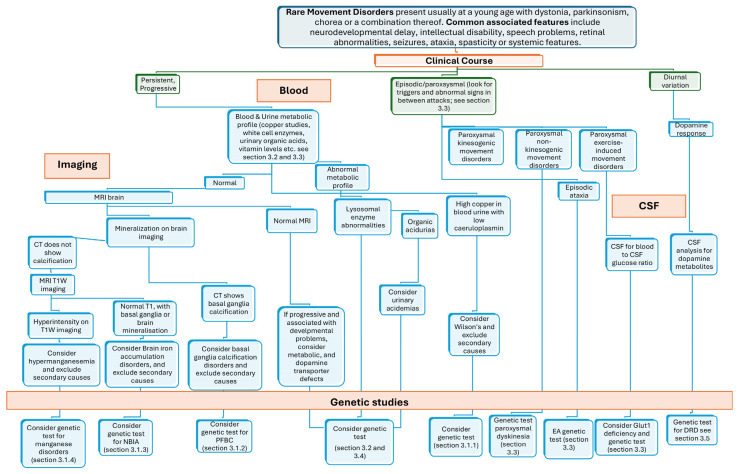
A decision-tree approach linking clinical, genetic, and imaging testing to facilitate the recognition of RMDs overviewed in this review. Legend: DRD, dopa-responsive dystonia; EA, episodic ataxia; NBIA, neurodegeneration with brain iron accumulation; PFBC, primary familial brain calcification.

Figure 1 illustrates a decision-tree approach that integrates clinical, neuroimaging, and laboratory data. It begins with the temporal pattern of motor symptoms and signs (persistent/progressive, episodic/paroxysmal, or showing diurnal variation) and progresses towards genetic testing, guided by neuroradiological and laboratory findings. This structured flowchart aims to support the diagnostic workup of the RMD reviewed in this article.

## 3. Overview and Phenotypic Clues of RMD

### 3.1. Brain Mineralopathies

#### 3.1.1. Copper

Wilson’s disease (WD) is the best-known brain mineralisation disorder and is caused by biallelic variants in *ATP7B*, which normally encodes a copper-transporting P-type ATPase expressed predominantly in the liver [34]. Defects in the encoded protein lead to reduced biliary excretion, excessive hepatic storage, and plasma accumulation of copper, which ultimately causes copper deposition in other organs, including the nervous system and eyes [35]. Manifestations of WD reflect the distribution of copper pathological deposition, with neurological features encompassing tremor, chorea, dystonia, dysautonomia, seizures, and psychiatric disturbances [36]. Clinical and ophthalmological assessments may importantly reveal Kayser–Fleischer rings, i.e., abnormal copper deposition within the Descemet membrane, which are pathognomonic of the disease [24]. Biochemical testing can be helpful, classically demonstrating low serum ceruloplasmin and copper, as well as high 24 h urinary copper levels [36]. A brain MRI may additionally reveal the “face of the giant panda” sign relating to the midbrain, in which the red nucleus and substantia nigra are surrounded by high T2 signals in the tegmentum [37]. Other possible signs that have been described include the “face of the miniature panda”, “double panda”, “bright claustrum”, “split thalamus”, and “whorl” signs [38,39,40]. WD is a treatable condition, and the outcome is very favourable in treated vs. untreated cases. The best evidence for treatment is by copper chelation, using D-penicillamine and trientine, to increase the urinary excretion of copper [6]. Zinc salts may also be used to prevent copper absorption in the gut, although they should not be used in conjunction with chelating agents [41]. In severe circumstances, liver transplantation may be required [36].

#### 3.1.2. Calcium

Idiopathic (or inherited) basal ganglia calcification (IBGC) disorders are diseases characterised by extensive, bilateral, symmetrical calcification in the basal ganglia that shows a coarse conglomerate pattern on imaging [42]. However, the dentate nuclei, thalamus, brainstem, centrum semiovale, and subcortical white matter may also be involved [43]. These disorders tend to present with a wide range of neurological and psychiatric features (Table 2). Until recently, IBGC was assumed to be inherited in an autosomal dominant fashion, justifying the term “primary familial brain calcification” [44]. However, the widespread availability of next-generation sequencing has also enabled the identification of BGC-related genes with an autosomal recessive pattern of inheritance (Table 2) [30]. The treatment of IBGC disorders is currently supportive and guided toward symptomatic management [44]. Furthermore, it should be noted that calcium deposition in the brain may not always be pathological, especially in the absence of clinical signs and symptoms and other secondary causes need to also be excluded in the differential diagnosis (e.g., hypoparathyroidism, infections of the central nervous system, human immunodeficiency virus, and systemic lupus erythematosus), which may possess more effective treatments [45].

#### 3.1.3. Iron

Neurodegeneration with brain iron accumulation (NBIA) represents a group of inherited disorders characterised by the abnormal accumulation of iron in the basal ganglia and, in some cases, the dentate nuclei (Table 3) [46]. The majority of these display an autosomal recessive inheritance pattern (PKAN, PLAN, MPAN, CoPAN, aceruloplasminemia, FAHN, KRD/PARK9, or WSS), although autosomal dominant (neuroferritinopathy) and X-linked dominant (BPAN) inheritance patterns have also been described [46,47]. In addition to the classical forms, novel NBIA syndromes have been described in the last decade [48]. There is significant variability in the age of onset between NBIA subtypes [46]. Clinical features of NBIA typically involve a progressive dystonia and dysarthria, spasticity, parkinsonism, neuropsychiatric disturbance, optic atrophy, and retinal degeneration [47]. Although Woodhouse–Sakati syndrome is often grouped with NBIA disorders due to overlapping neuroimaging findings, it is more accurately characterized as a multisystem endocrinopathy. In addition to neurological symptoms, it typically presents with hypogonadism, diabetes mellitus, hypothyroidism, and progressive childhood-onset alopecia [49]. The treatment of NBIA is also largely guided towards symptomatic management, utilising pharmacological agents or, in selected cases, deep brain stimulation [50]. An exception to this is noted for aceruloplasminaemia, for which iron chelation and plasma-derived ceruloplasmin supplementation have proven themselves as effective treatment strategies to diminish central nervous system iron accumulation and prevent/ameliorate associated neurological symptoms [51].

#### 3.1.4. Manganese

Genetically determined hypermanganesemia, leading to manganese accumulation in the brain, also accounts for a group of RMDs. Mutations in *SLC30A10* and *SLC39A14* have been recently described to lead to hypermanganesemia [52,53]. A further gene, *SLC39A8*, has also been linked to manganese homoeostasis; however, it codes for a transporter protein responsible for the cellular uptake of manganese, and hence, mutations lead to decreased manganese levels within the brain [54]. The clinical presentation of these disorders usually accompanies parkinsonism; however, gait impairment and postural instability tend to occur earlier than Parkinson’s Disease [55]. The gait abnormality in hypermanganesemia additionally appears to be characterised by retropulsion, propulsion, freezing, and a characteristic ”cock-walk” (the individual walks on the metatarsophalangeal joints associated with an erect spine and flexed elbows) [56]. Dystonia and neuropsychiatric changes have also been described [55].

The genetic forms of hypermanganesemia can be identified early, presenting in childhood or early adolescence. Patients with *SLC30A10* mutations tend to present slightly earlier (often in infancy) than those with *SLC39A14* mutations, have slightly higher blood manganese levels and also exhibit manganese content in the muscle [57,58]. MRI is invaluable in manganese deposition disorders, demonstrating hyperintense signal on T1-weighted imaging in the globus pallidi bilaterally [33], whilst T2 sequences show normal signal in these corresponding areas [59]. Additional investigations may also be useful, particularly in differentiating between the genetic forms, as hepatomegaly/cirrhosis with an associated transaminitis or polycythaemia are commonly reported in patients with *SLC30A10* mutations, but not *SLC39A14* mutations [57]. The treatment of hypermanganesemia is primarily conducted with chelation therapy using intravenous disodium calcium edetate, which increases the urinary excretion of manganese to result in reduced serum levels [60].

### 3.2. Lysosomal Storage Diseases

Lysosomal storage diseases (LSDs) are inherited metabolic disorders that result in the progressive accumulation of substrates, leading to damage across multiple tissues [61]. These diseases typically manifest in childhood, with the central nervous system frequently being involved [62]. Examples of LSDs include Gaucher’s Disease, Fabry Disease, Niemann–Pick Disease Type C, Neuronal Ceroid Lipofuscinoses, Mucopolysaccharidoses, aspartylglucosaminuria, Salla disease, and Tay–Sachs disease [61]. The clinical presentation of LSDs can vary widely (Table 4), although ataxia is often a common symptom, typically accompanied by dystonia, myoclonus, or a resting tremor [61]. While many patients are managed with supportive care, enzyme replacement therapy has proven to be an effective treatment for certain LSDs, compensating for the defective metabolic process [63,64,65]. Substrate reduction therapy is another potential treatment approach, and it may be more effective when combined with enzyme replacement therapy; however, it is currently approved only for a limited number of LSDs [65,66]. LSDs often result from missense mutations, which lead to abnormal folding of the lysosomal enzymes, preventing their normal function; hence, chaperone therapy denotes another treatment measure by which administered “chaperone” molecules assist the proteins to fold correctly and regain at least part of their catalytic activity [65]. Haematopoietic stem cell transplantation, especially used prior to the development of enzyme replacement therapy [65], allows donor cells to migrate into the recipient’s organs and correct the metabolic defect by locally releasing the missing enzyme. Gene therapy and stop-codon readthrough may be further avenues of treatment to correct the underlying pathological mutation of LSDs, but these require further study to validate their use [65].

### 3.3. Episodic Movement Disorders

Paroxysmal movement disorders are characterised by episodic involuntary movements and are largely divided into paroxysmal dyskinesia or episodic ataxia (EA) [67]. The pathophysiological framework of paroxysmal movement disorders revolves around abnormalities in ion channels, proteins of the vesical synaptic cycle, or proteins implicated in neuronal energy metabolism [67]. Symptoms often begin in childhood and may improve with age, although with episodic ataxia, symptoms can also worsen [68].

Paroxysmal dyskinesia typically presents with transient episodes of involuntary movements, particularly dystonia and/or chorea, without loss of consciousness [69]. Paroxysmal dyskinesia is further subdivided into paroxysmal kinesigenic dyskinesia (PKD), paroxysmal non-kinesigenic dyskinesia (PNKD) and paroxysmal exercise-induced dyskinesia (PED), dependent on the underlying trigger of the episodes [70]. Mutations in the *SLC2A1* gene, which encodes the glucose transporter GLUT1, have been particularly linked to a phenotypic spectrum of isolated PED [71]. The last two decades have additionally provided much greater insight into the aetiology of PKD, establishing associations with mutations in the *PRRT2*, *TMEM151A*, and *KCNJ10* genes [72,73,74,75]. The *PRRT2* gene, which encodes a protein that interacts with the SNAP25 protein that affects presynaptic neurotransmitter release, accounts for the large majority of familial cases of PKD and also a significant proportion of sporadic cases [76,77]. The pathophysiology of PNKD remains unclear, but it is suggested to be linked to mutations in the *PNKD* gene, as this has been reported in up to 60% of patients [78]. Episodic ataxia (EA), on the other hand, presents with episodes of cerebellar dysfunction, typically accompanied by dystonia, hemiplegia, headaches, or tinnitus [79]. Unlike paroxysmal dyskinesia, EA is subdivided by the underlying genetic abnormality (e.g., *KCNA1* mutations in EA1, *CACNA1A* mutations in EA2), as there is often significant overlap in clinical characteristics between the different subtypes [79]. Examination during the interictal phase of paroxysmal movement disorders is usually normal; however, in EA, nystagmus or myokymia may be present [80]. Requesting video recordings of the episodes is therefore essential to facilitate early diagnosis.

The treatment of paroxysmal movement disorders is primarily focused on managing symptoms and preventing attacks. A combination of supportive measures and therapeutic medications is subsequently used. The avoidance of triggers (e.g., emotional stress, fatigue, and alcohol/caffeine consumption) is pivotal and may be highly effective in preventing attacks [81]. A ketogenic diet is also recommended in PED, as it provides an alternative energy source to bypass the defective GLUT1 protein [82,83]. Potential medications include anticonvulsants, benzodiazepines, levodopa, and acetazolamide. Anticonvulsants are particularly efficacious in PKD, whilst benzodiazepines tend to be more helpful in PNKD [84,85]. In contrast, EA2 responds very well to acetazolamide and 4-aminopyridine [86].

### 3.4. Vitamin Deficiency-Related Disorders

A genetically determined inability to produce or utilise vitamins can also lead to RMDs. These diseases include biotinidase deficiency (*BTD* mutations), biotin–thiamine-responsive basal ganglia disease (*SLC19A3* mutations), abetalipoproteinemia (*MTTP* mutations), ataxia with vitamin E deficiency (*TTPA* mutations), homocystinuria (*CBS* mutations), cobalamin deficiency, cerebral folate deficiency (*FLR1* and *SLC46A1* mutations), coenzyme Q10 deficiency, and pyruvate dehydrogenase complex deficiency [87]. Symptoms vary significantly between vitamin deficiency-related disorders, although patients typically present with ataxia, dystonia, parkinsonism, or spasticity (Table 4) [87].

MRI can be useful in these disorders. For instance, biotin–thiamine-responsive basal ganglia disease can show bilateral high T2 signal intensity in the caudate head, putamen, globi pallidi, thalami, infra- and supra-tentorial cortex, brainstem, and cerebellum [88]. However, other disorders, such as cerebral folate deficiency, can appear normal on MRI, although they more typically demonstrate diffuse, leukodystrophy-like, white matter changes [89]. The treatment of vitamin deficiency-related disorders involves replacing the missing or impaired vitamins. Additional dietary measures may also be implemented, such as a ketogenic diet for pyruvate dehydrogenase complex deficiency, which produces a significant improvement in symptoms [6,90].

### 3.5. Dopamine Responsive Disorders

Dopa-responsive dystonia (DRD) represents a group of disorders, typically manifesting with limb-onset, diurnally fluctuating dystonia (particularly commencing in childhood) that exhibit a robust and sustained response to levodopa treatment [91]. These disorders are often caused by genetic defects in the biosynthesis of dopamine, demonstrating a wide spectrum of clinical manifestations (Table 5). However, DRD has also seldom been associated with conditions that do not affect dopamine biosynthesis pathways (e.g., spinocerebellar ataxia type 3, ataxic telangiectasia, and hereditary spastic paraplegia type II) [91,92]. There has subsequently been a recent suggestion from Lee et al. (2018) for the definition of DRD to be refined to represent a group of non-neurodegenerative conditions with genetic defects involving the nigrostriatal dopaminergic system with cardinal manifestations [93]. They additionally posed a further group of conditions, DRD-plus disorders, which appear similar to DRD but also present with atypical features, such as hypotonia, drowsiness, ptosis, cerebellar dysfunction, infantile onset, developmental delay, psychomotor retardation, and seizures [93]. Finally, a third group, DRD look-alikes, was also suggested, which encompasses neurodegenerative (e.g., ataxia telangiectasia) or non-neurodegenerative (e.g., *TOR1A*-related dystonia, GLUT1 deficiency syndrome, myoclonus–dystonia) conditions that do not involve the nigrostriatal dopaminergic system, or neurodegenerative disorders that involve the nigrostriatal dopaminergic system (e.g., juvenile Parkinson’s disease, pallidopyramidal syndrome, and spinocerebellar ataxia type 3) [93].

## 4. Further Treatment Considerations

### 4.1. Conservative Treatments

Education and counselling play a crucial role in the management of RMDs, directing patients and their carers toward reliable web-based resources and support groups that are increasingly accessible. The involvement of multidisciplinary teams, particularly occupational therapists, physiotherapists, and speech and language therapists, provides the opportunity to support and empower patients in adapting their lifestyle around their disorder. As previously outlined, dietary modifications and trigger avoidance can be highly effective and straightforward interventions in certain disorders. However, in refractory cases, clinicians should remain vigilant to the possibility of patient non-compliance to these measures. Additionally, managing patients with RMDs involves screening for known complications associated with the disorder, such as malignancy in ataxic telangiectasia [94].

### 4.2. Medical Treatments

It is important to recognise that, although various medical (mostly symptomatic) treatments are available for RMDs, clinical trials assessing their efficacy and safety often involve small sample sizes and lack long-term outcome data [6]. Despite these limitations, due to the substantial impact of these disorders on quality of life, it is common practice for clinicians to prescribe such treatments off-label, while closely monitoring for possible therapeutic complications. Furthermore, in the context of RMDs, certain disorders may be highly responsive to specific therapies, potentially leading to complete symptom remission [6]. Therefore, the early recognition of treatable RMDs is crucial for optimising patient outcomes (Table 6).

### 4.3. Genetic Testing and Counselling

The early recognition of RMDs can be significantly facilitated by the advances and wide availability of next-generation sequencing, as well as constantly improving pipelines for data analysis, which are increasingly integrated into the standard diagnostic workflow for these conditions [95]. For instance, a recent study involving a wide spectrum of ataxia phenotypes identified a genetic basis in 50% of the cohort using whole-exome sequencing [96]. Moreover, molecular testing plays a crucial role in elucidating the phenotypic heterogeneity of genetic RMDs, as exemplified by *GNAO1*-related disorders that are now known to present with both infantile dyskinetic encephalopathy and late-onset focal abnormal movements [97,98]. However, both whole-exome and whole-genome sequencing generate vast amounts of data, posing a challenge in determining which identified variants are causally linked to the phenotype under investigation [95]. Automated pipelines, using a series of computational mutation-filtration steps, partly address this challenge, but these depend heavily on online resources and bioinformatics tools, which still have considerable limitations. As a result, several variants of uncertain significance remain, and there is ongoing debate regarding whether these findings should be reported and communicated to patients and their families [95]. The re-analysis of genomic sequencing data at regular intervals will be crucial to address this challenge. In addition, sharing findings from molecular testing within the broader genetics community will help to strengthen genotype–phenotype correlations. This is especially pertinent in light of recent advances in gene therapy, which present a promising therapeutic approach for RMDs, underscored by the initiation of the first clinical trial utilising gene therapy for AADC deficiency [99].

The molecular diagnosis of RMDs has significant implications for genetic counselling, as it allows for a more accurate assessment of the inheritance pattern and potential risk for family members of affected individuals. Identifying the underlying genetic cause can provide crucial information for relatives regarding their own risk of developing the disorder, as well as the likelihood of passing it on to future generations. This information can also guide family planning, including prenatal testing and carrier screening. Additionally, genetic counselling can help families understand the implications of the diagnosis, manage expectations regarding disease progression, and make informed decisions about available treatment options and participation in clinical trials.

## 5. Conclusions

Although rare by definition, RMDs as a group are often encountered by movement disorder specialists, requiring a detailed clinical history and examination as the first step to narrow down the differential diagnosis. Investigations, including biochemical tests, neuroimaging, and genetic tests, can be crucial in elucidating the etiological diagnosis. Certain RMDs respond very well to specific therapies, sometimes resulting in complete symptom remission, making it essential not to overlook these conditions. Collaborative efforts should continue to enhance the understanding of RMDs, particularly with regard to genotype–phenotype correlations, and further develop RMD patient registries and biobanks to facilitate ongoing research. Advances in dissecting the molecular basis of RMDs offer a promising avenue for treatment with gene therapy, and this may emerge as an effective management option for several of these disorders in the future.

## Figures and Tables

**Table 1:** Clinical cues and helpful investigation findings, suggestive of a specific diagnosis.

RMD Category	Diagnostic Clues and Relevant Investigations	Examples of Specific Conditions
Dopamine synthesis disorders	Oculogyric crises	TH deficiency, AADC deficiency Sepiapterin deficiency
	Diurnal fluctuation of symptom severity Oculogyric crises	GCH deficiency (classic DRD—Segawa disease)
Disorders with brain mineralisation	Kayser-Fleischer ring Sunflower cataract Low serum ceruloplasmin High 24 h urinary copper level Face of the giant panda sign on MRI	Wilson’s disease
	Retinal degeneration/optic atrophy Retinitis pigmentosa Mantis sign Eye of the tiger sign on MRI	NBIA (PKAN and VAC14) (PKAN) (PKAN)
Metabolic diseases	Vertical supranuclear gaze palsy (particularly downward) High plasma oxysterol levels	NP-C
	Cherry-red spot on the fundus Low serum hexosaminidase A	Tay–Sachs disease
	Tendon xanthomas	Cerebrotendinous xanthomatosis
Paroxysmal MD	Exercise-induced dyskinesia Low CSF-to-blood glucose ratio CSF lactate low to normal	PED (GLUT1 deficiency syndrome)
	Dyskinesia (variable frequency of attacks, may be up to 100 per day, lasting <1 min) mainly triggered by sudden movement from rest	PKD
	Dyskinesia (1–3 attacks per day lasting minutes to hours) sometimes triggered by alcohol, caffeine, stress, menstruation, or sleep deprivation	PNKD
	Interictal myokymia	EA1

Legend: AADC, aromatic L-amino acid decarboxylase; DRD, dopa-responsive dystonia; EA1, episodic ataxia type 1; GCH, GTP cyclohydrolase; MD, movement disorders; NBIA, neurodegeneration with brain iron accumulation; NP-C, Niemann–Pick disease type C; PED, paroxysmal exercise-induced dyskinesia; PKAN, pantothenate kinase-associated neurodegeneration; PKD, paroxysmal kinesigenic dyskinesia; PNKD, paroxysmal non-kinesigenic dyskinesia; TH, tyrosine hydroxylase.

**Table 2:** Causative genes associated with primary familial brain calcification disorders and common clinical features.

Causative Genes	Inheritance Pattern	Phenotypic Features
*SLC20A2*	Autosomal dominant	*Movement disorders:* Bradykinesia, rigidity, tremor, dystonia, ataxia, chorea, pyramidal *Cognitive/psychiatric features:* Cognitive deficits, depression, psychosis, anxiety *Other symptoms/signs:* Headache, speech disturbance, seizures, dysmorphic features *Imaging:* Basal ganglia calcification along with calcifications in the cerebellum (dentate nucleus), white matter, pons
*PDGFB*	Autosomal dominant	
*PDGFRB*	Autosomal dominant	
*XPR1*	Autosomal dominant	
*JAM2*	Autosomal recessive	
*MYORG*	Autosomal recessive	
*NAA60*	Autosomal recessive	

**Table 3:** Clinical and specific neuroradiological features of NBIA subtypes.

Disease and Causative Gene	Specific Clinical Features (Alongside Chorea, Dystonia, and Parkinsonism)	Potential MRI Findings
PKAN *PANK2*	Oromandibular and truncal extensor dystonia (opisthotonus)	Eye of the tiger sign in the globus pallidus Mid-hypointensity in substantia nigra
PLAN *PLA2G6*	Axial hypotonia (in early-childhood onset) Opisthotonus (in late-childhood onset) Seizures Children and young adults may also present with ataxia, speech regression, optic atrophy, neuropsychiatric disturbance, oculomotor abnormalities, and autonomic disturbance	Cerebellar and vermian atrophy with callosal thinning, vertical orientation, and claval hypertrophy
NEUROFERRITINOPATHY *FTL*	Triad of oromandibular dyskinesia, dysarthrophonia and action-specific facial dystonia	Cortical pencil sign Hypointense caudate, putamen, thalamus, globus pallidus, substantia nigra, and red nucleus
MPAN *C19orf12*	Often presents in childhood with spastic paraparesis, behavioural disturbance, optic atrophy, and motor axonal neuropathy	Hypointensity in the globus pallidus and substantia nigra Hyperintense streaking of the medial medullary lamina of the globus pallidus Cortical and cerebellar atrophy
BPAN *WDR45*	Biphasic disease, presenting in childhood with delayed speech and motor development, ataxic gait Cognitive decline and seizures in adulthood	Halo sign in substantia nigra on T1 Hyperintensity on T2 in the globus pallidus and cerebral peduncles
CoPAN *COASY*	Spasticity, axonal neuropathy, cognitive decline, and obsessive–compulsive behaviour in childhood	Eye of the tiger sign Hypointense substantia nigra and globus pallidus Swelling of the caudate and putamen
ACERULOPLASMINEMIA *CP*	Triad of retinal degeneration, diabetes mellitus, and anaemia Cerebellar dysfunction	Hypointense caudate, putamen, thalamus, globus pallidus, substantia nigra, cerebellum, and red nucleus
FAHN *FA2H*	Presents in childhood with cerebellar dysfunction, spasticity, optic atrophy, bristly hair, and seizures	Globus pallidus more hypointense than substantia nigra Confluent subcortical and periventricular cerebral T2 white matter hyperintensities Atrophy of cerebellum, medulla, and spinal cord
KRD/PARK9 *ATP13A2*	Presents in adolescence with vertical supranuclear gaze palsy (particularly upward), facial-faucial–finger myoclonus and visual hallucinations	Diffuse cerebral, cerebellar, and brainstem atrophy Iron accumulation in putamen and caudate
WSS *DCAF17*	Dysarthria, dysphagia, seizures, sensory polyneuropathy, and sensorineural deafness Craniofacial abnormalities and camptodactyly Alopecia, hypogonadism, hypothyroidism, and diabetes mellitus	Partially empty sella Progressive white matter lesions in the frontoparietal region Iron accumulation in the globus pallidus, substantia nigra, red nucleus

Legend: BPAN, beta-propeller protein-associated neurodegeneration; CoPAN, COASY protein-associated neurodegeneration; FAHN, fatty acid 2-hydroxylase deficiency; KRD, Kufor Rakeb disease; MPAN, mitochondrial membrane protein-associated neurodegeneration; PKAN, pantothenate kinase-associated neurodegeneration; PLAN, PLA2G6-associated neurodegeneration; WSS, Woodhouse–Sakati syndrome.

**Table 4:** Clinical features of lysosomal storage disorders, vitamin deficiency-related disorders, and other metabolic diseases.

Disease	Exemplar Neurological Features
Abetalipoproteinemia	Tremor, nystagmus, peripheral neuropathy, delayed intellectual development, loss of deep tendon reflexes, retinitis pigmentosa
Aspartylglucosaminuria	Slowly progressive clumsiness, speech delay, hyperkinesia, facial dysmorphism
Ataxia with vitamin E deficiency	Progressive cerebellar syndrome, areflexia, positive Babinski sign, macular degeneration, pigmentary retinopathy
Biotinidase deficiency	Ataxia, seizures, hypotonia, developmental delay, optic atrophy, sensorineural hearing loss
Biotin-thiamine responsive basal ganglia disease	Recurrent subacute encephalopathy with confusion, seizures, ataxia, dystonia, cogwheel rigidity, supranuclear facial palsy, external ophthalmoplegia
Cerebral folate deficiency	Irritability, sleep disturbance, psychomotor retardation, dyskinesia, cerebellar ataxia, spastic diplegia, visual disturbance, sensorineural hearing loss
Cobalamin deficiency	Myelopathy, peripheral neuropathy with abnormal proprioception, cognitive impairment, dysesthesia, spastic paraparesis or tetraparesis, optic nerve atrophy
Coenzyme Q10 deficiency	Encephalopathy, seizures, dystonia, spasticity, intellectual disability, hypotonia, autonomic dysfunction, parkinsonism, cerebellar ataxia, pyramidal dysfunction, peripheral neuropathy
Glutaric acidaemia type 1	Progressive macrocephaly, hypotonia, motor regression, seizures, headaches, vertigo, ataxia, dementia
Homocystinuria	Developmental delay, autism spectrum disorders, psychiatric disturbance, marfanoid appearance, ectopia lentis
Methylmalonic aciduria	Encephalopathy, failure to thrive, hypotonia, developmental delay, ataxia, dysarthria, spastic paraparesis, seizures
Phenylketonuria	Microcephaly, epilepsy, severe developmental delay, progressive supranuclear motor disturbances, tremor, cerebellar ataxia
Propionic acidaemia	Progressive encephalopathy, seizures, coma, hypotonia, developmental delay
Fabry Disease	Hemiparesis, diplopia, headaches, cerebellar syndrome, dysmetria, neuropsychiatric disturbance, dementia
Gaucher’s Disease	Oculomotor apraxia, seizures, progressive myoclonic epilepsy, bulbar and/or pyramidal signs, cognitive impairment
Mucopolysaccharidoses	Developmental delay, neurocognitive regression, behavioural changes, sleep disturbance, epilepsy, hydrocephalus, raised intracranial pressure symptoms, myelopathy
Neuronal Ceroid Lipofuscinoses	Progressive mental and motor deterioration, retinal degeneration causing blindness, epilepsy
Niemann–Pick Disease Type C	Hypotonia, developmental delay, vertical supranuclear gaze palsy, ataxia, seizures, gelastic cataplexy, dystonia, spasticity
Pyruvate dehydrogenase complex deficiency	Developmental delay and motor regression, growth retardation, hypotonia, seizures, ataxia, dystonia
Salla disease	Psychomotor delays, spasticity, athetosis, epileptic seizures, hypotonia, cerebellar syndrome
Tay–Sachs disease	Hypotonia, developmental delay and motor regression, decreased responsiveness over time, spasticity, seizures, ataxia, dyskinesia, sleep disturbance, screaming and irritability, macrocephaly, decerebrate posturing, dysphagia, dystonia, cognitive impairment

**Table 5:** Clinical features and useful biochemical investigations for common disorders associated with a defect in the dopamine synthesis pathway.

Disorder	Enzyme/Protein Deficiency	Clinical Features	Catecholamine Metabolite Levels
Biopterin synthesis/recycling defects	SPR	Dystonia, weakness, oculogyric crisis, axial hypotonia, motor, and speech delay	↓ HVA ↓ 5-HIAA
	AD-GTPCH1	Gait disturbance due to foot dystonia with later development of generalised dystonia and parkinsonism	N/↓ HVA N/↓ 5-HIAA
	AR-GTPCH1		↓ HVA ↓ 5-HIAA
	PTPS	Developmental delay, axial hypotonia, hypertonia of extremities, small for gestational age	↓ HVA ↓ 5-HIAA
	DHPR	Developmental delay, axial hypotonia, hypertonia of extremities, epilepsy, microcephaly	↓ HVA ↓ 5-HIAA
	PCD	Progressive hypotonia and delays in motor development, which are temporary	↓ HVA ↓ 5-HIAA
Primary neurotransmitter synthesis defects	TH	Gait disturbance due to lower-limb dystonia, which gradually progresses to generalised dystonia +/− parkinsonism with pyramidal signs	↓ HVA N 5-HIAA
Monoamine transportopathies	DTDS *SLC6A3*	Chorea, dystonia, ballismus, and orolingual dyskinesia, which progresses to involve parkinsonism–dystonia	↑ HVA N 5-HIAA
	VMAT2 *SLC18A2*	Motor delay, eye deviation, severe parkinsonism, dystonia, ataxia, oculogyric crises, autonomic dysfunction and ptosis	N HVA N 5-HIAA
Monoamine catabolism disorders	MAO-A/ MAO-B	Episodic hypotonia, intellectual disability, stereotyped movements, aggressive behaviour.	↓ HVA ↓ 5-HIAA
	DBH	Autonomic dysfunction, reduced exercise tolerance, hypoglycaemia, and ptosis	↑ HVA ↑ 5-HIAA
	AADC	Truncal hypotonia, oculogyric crises, hypokinesia, athetosis, chorea, tremor, autonomic dysfunction	↓ HVA ↓ 5-HIAA
	PNPO	High seizure frequency, developmental delay, hypotonia, dystonia	↓ HVA ↓ 5-HIAA

Legend: AADC, aromatic L-amino acid decarboxylase; AD-GTPCH1, autosomal dominant guanosine-triphosphate cyclohydrolase 1; AR-GTPCH1, autosomal recessive guanosine-triphosphate cyclohydrolase 1; DBH, dopamine beta-hydroxylase; DHPR, dihydropteridine reductase; DTDS, dopamine transporter deficiency syndrome; MAO-A, monoamine oxidase A; MAO-B, monoamine oxidase B; N, normal; PNPO, pyridoxamine 5′-phosphate oxidase; PTPS, 6-pyruvoyl-tetrahydropterin synthase; SPR, sepiapterin reductase; TH, tyrosine hydroxylase; VMAT2, vesicular monoamine transporter 2; ↓, low levels; ↑, elevated levels.

**Table 6:** Treatable rare movement disorders.

Rare Movement Disorder	Treatment
Abetalipoproteinaemia	Dietary fat restriction, vitamin E and A
Aromatic L-amino acid decarboxylase deficiency	Dopamine agonists, monoamine oxidase inhibitors, pyridoxine
Ataxia with vitamin E deficiency	Vitamin E
Biotin–thiamine responsive basal ganglia disease	Biotin plus thiamine, avoid or treat triggers
Biotinidase deficiency	Biotin
Cerebral creatine deficiency	Creatine ± ornithine, dietary restriction of arginine
Cerebral folate deficiency	Folinic acid
Cerebrotendinous xanthomatosis	Chenodeoxycholic acid
Cobalamin deficiency	Cobalamin derivatives
Coenzyme Q10 deficiency	Coenzyme Q10
Dopa-responsive dystonia	Levodopa, 5-hydroxytryptophan
Dystonia–parkinsonism with manganese accumulation	EDTA chelation therapy
Episodic ataxia type 2	4-aminopyridine, acetazolamide
Gaucher disease	Enzyme replacement therapy, N-butyl-deoxynojirimycin
GLUT1 deficiency syndrome	Ketogenic diet, triheptanoin
Glutaric aciduria type 1	Avoid or treat triggers, dietary lysine restriction, L-carnitine
Homocystinuria	Vitamin B6, dietary restriction of methionine, betaine
Maple syrup urine disease	Avoid or treat triggers, dietary leucine restriction
Methylmalonic aciduria	Avoid or treat triggers, dietary protein restriction, L-carnitine
Molybdenum cofactor deficiency	Cyclic pyranopterin monophosphate
Niemann–Pick type C	Miglustat (N-butyl-deoxynojirimycin)
Paroxysmal kinesigenic dyskinesia	Carbamazepine, other anticonvulsants
Phenylketonuria	Dietary restriction of phenylalanine
Propionic acidaemia	Avoid or treat triggers, dietary protein restriction, L-carnitine
Pyruvate dehydrogenase complex deficiency	Thiamine, ketogenic diet, triheptanoin
Refsum disease	Dietary restriction of phytanic acids, plasmapharesis
Wilson’s disease	Penicillamine, trientine, zinc

## Data Availability

No new data were generated within this work.
